# Myopia control in Mendelian forms of myopia

**DOI:** 10.1111/opo.13115

**Published:** 2023-03-07

**Authors:** Emilie van der Sande, Jan Roelof Polling, J. Willem L. Tideman, Magda A. Meester-Smoor, Alberta A. H. J. Thiadens, Emily Tan, Chris I. De Zeeuw, Ralph Hamelink, Ingo Willuhn, Virginie J. M. Verhoeven, Beerend H. J. Winkelman, Caroline C. W. Klaver

**Affiliations:** 1 Department Ophthalmology, Erasmus Medical Center, Rotterdam, The Netherlands; 2 Department Epidemiology, Erasmus Medical Center, Rotterdam, The Netherlands; 3 Netherlands Institute for Neuroscience, Amsterdam, The Netherlands; 4 Departments Orthoptics and Optometry, Hogeschool Utrecht, Utrecht, The Netherlands; 5 Department Ophthalmology, Martini Hospital, Groningen, The Netherlands; 6 Department Neuroscience, Erasmus Medical Center, Rotterdam, The Netherlands; 7 Department Psychiatry, Amsterdam University Medical Centers, Amsterdam, The Netherlands; 8 Department Clinical Genetics, Erasmus Medical Center, Rotterdam, The Netherlands; 9 Department Ophthalmology, Radboud Medical Center, Nijmegen, The Netherlands; 10 Institute of Molecular and Clinical Ophthalmology, Basel, Switzerland

**Keywords:** atropine, dopamine, *lipoprotein receptor-related protein 2* knockout mouse, Mendelian myopia, myopia control, syndromic myopia

## Abstract

**Purpose:**

To study the effectiveness of high-dose atropine for reducing eye growth in Mendelian myopia in children and mice.

**Methods:**

We studied the effect of high-dose atropine in children with progressive myopia with and without a monogenetic cause. Children were matched for age and axial length (AL) in their first year of treatment. We considered annual AL progression rate as the outcome and compared rates with percentile charts of an untreated general population.

We treated C57BL/6J mice featuring the myopic phenotype of Donnai–Barrow syndrome by selective inactivation of *Lrp2* knock out (KO) and control mice (CTRL) daily with 1% atropine in the left eye and saline in the right eye, from postnatal days 30–56. Ocular biometry was measured using spectral-domain optical coherence tomography. Retinal dopamine (DA) and 3,4-dihydroxyphenylacetic acid (DOPAC) were measured using high-performance liquid chromatography.

**Results:**

Children with a Mendelian form of myopia had average baseline spherical equivalent (SE) –7.6 ± 2.5D and AL 25.8 ± 0.3 mm; children with non-Mendelian myopia had average SE −7.3 ± 2.9 D and AL 25.6 ± 0.9 mm. During atropine treatment, the annual AL progression rate was 0.37 ± 0.08 and 0.39 ± 0.05 mm in the Mendelian myopes and non-Mendelian myopes, respectively. Compared with progression rates of untreated general population (0.47 mm/year), atropine reduced AL progression with 27% in Mendelian myopes and 23% in non-Mendelian myopes*.*

Atropine significantly reduced AL growth in both KO and CTRL mice (male, KO: −40 ± 15; CTRL: −42 ± 10; female, KO: −53 ± 15; CTRL: −62 ± 3 μm). The DA and DOPAC levels 2 and 24 h after atropine treatment were slightly, albeit non-significantly, elevated.

**Conclusions:**

High-dose atropine had the same effect on AL in high myopic children with and without a known monogenetic cause. In mice featuring a severe form of Mendelian myopia, atropine reduced AL progression. This suggests that atropine can reduce myopia progression even in the presence of a strong monogenic driver.

**Supplementary Information:**

The online version of this article (doi:10.1111/opo.13115) contains supplementary material, which is available to authorized users.

## Key points

- High-dose atropine reduced axial length progression in highly myopic children with and without a known monogenetic cause.
- Atropine reduced axial length progression in mice featuring a severe form of Mendelian myopia.
- Dopamine and DOPAC levels were slightly, albeit non-significantly, elevated after atropine treatment.

## INTRODUCTION

Myopic refractive errors, particularly in the more extreme values, can lead to ocular complications later in life. The risk of visual loss is strongly related to axial length (AL), such that ALs of 30 mm or more have a >90% risk of developing severe visual impairment.^1^ The majority of common myopia has a complex genetic origin; >500 genetic loci have currently been identified for this trait.^2,3^ Myopia can also be inherited in a Mendelian fashion by a single mutation in one causal gene. Examples of such inheritance are the *FBN1* gene causing Marfan syndrome, *COL2A1* leading to Stickler syndrome, *CACNA1F* involved in congenital stationary night blindness and *LRP2* responsible for Donnai–Barrow syndrome.^4–8^ These genes often cause very high levels of myopia. Similar to non-Mendelian myopia, higher levels of myopia increase the risk of severe visual impairment in these syndromes.^9,10^

Current interventions for controlling myopia in children include optical and pharmacological therapies.^11^ High-dose atropine has proven to be most effective against myopia progression.^12,13^ This drug is a non-specific muscarinic receptor antagonist conventionally used for its mydriatic and cycloplegic effect. The mechanism underlying eye growth inhibition is not well understood, but regulation of dopaminergic activity has been proposed.^14–16^ In children with a non-Mendelian form of myopia, the response appears to be robust, as 86% showed a diminished progression rate of refractive error (RE) after 3 years of high-dose atropine therapy.^17^ Whether children with a strong monogenic driver for myopia also benefit from atropine therapy is currently unknown.

The aim of the current study was to test whether atropine treatment is effective in reducing eye growth in Mendelian forms of myopia. We investigated this in children with ophthalmogenetic disorders due to a mutation in a single gene, and in a mouse model with eye-specific loss of the *low-density lipoprotein receptor-related protein 2* (*Lrp2*) gene responsible for Donnai–Barrow syndrome in humans. Since its mechanism of action is still largely unresolved, we additionally tested whether atropine modulates downstream effects on dopaminergic activity in the retina.

## MATERIALS AND METHODS

The design of the children study was prospective and clinic-based. The setting was Erasmus Medical Center in Rotterdam, the Netherlands, and all consecutive children younger than 18 years of age presenting with progressive myopia were eligible. The study adhered to the tenets of the Declaration of Helsinki and was approved by the Institutional Review Board of Erasmus Medical Center. Eligible children and parents received a patient information leaflet followed by oral consultation, and participants provided written informed parental consent (for children >12 years of age: parents or legal guardians as well as the child; for children <12 years of age: only parents and legal guardians). The study population included two cohorts of children with progressive myopia on high-dose (0.5% or 1%) atropine treatment; AL growth rates for the first year of treatment were compared with percentiles in the growth curves of uncontrolled myopia.

### Mendelian myopic children on atropine treatment

This cohort consisted of 14 highly myopic children with a Mendelian ophthalmogenetic disorder on high-dose atropine treatment. Of these, 10 children were genetically diagnosed with a retinal dystrophy: congenital stationary night blindness (CSNB; genes *CACNA1F n* = 3, *NYX n* = 1); Bornholm disease (L/M opsin *n* = 2); retinitis pigmentosa (*RPGR n* = 3); oculocutaneous albinism (*TYR n* = 1). Three children were diagnosed with a connective tissue disorder: Marfan (*FBN1 n* = 1), Stickler (*COL2A1 n* = 2), and one child with a genetic mutation in a gene in the rod phototransduction cascade (*ARR3 n* = 1). Children were between 2 and 13 years old and had very long ALs, above the 93rd percentile and in some cases far beyond the 99th percentile of the European AL population growth chart.^18^

### Non-mendelian myopic children on atropine treatment

The second cohort served as a reference and consisted of highly myopic children without any clinical suspicion for a Mendelian disorder or any other pathology, who were also on atropine treatment. Data were derived from the Erasmus Medical Center myopia database, consisting of 512 children.^12^ Children with amblyopia, strabismus, systemic disorders and ex-premature children were excluded. We matched each Mendelian myopic child with non-Mendelian children from the database based on baseline age (±1 year), AL (±2 mm) and atropine dose (0.5% or 1%), which resulted in a reference cohort of 36 non-Mendelian myopes.

### Ophthalmological examination and atropine treatment

Participants underwent a standardised ophthalmological examination at baseline and at 6 and 12 months. At examination, cycloplegia was achieved using two drops of cyclopentolate 1% given 5 min apart, with a minimum waiting time of 45 min after the first instillation. An additional drop of cyclopentolate was applied if the iris was very dark and the pupil diameter <6 mm. RE was measured using a Topcon auto refractor (KR8900, global.topcon.com). Average RE per eye was calculated with at least three measurements. Spherical equivalent refraction (SE) was calculated as the average (sphere + 1/2 cylinder) of both eyes. AL was measured with the IOL Master (Carl Zeiss MEDITEC IOLMaster 700, zeiss.com). Five measurements per eye were used to calculate average AL of both eyes. Parents and participants were instructed to treat with high-dose atropine eye drops (0.5% or 1% atropine formulary Dutch pharmacies [FNA]) in both eyes daily before bedtime. All dosages were distributed in multi-dose bottles preserved with benzalkonium chloride, sodium edetate, boric acid and purified water.

### Rates of axial length progression in uncontrolled myopic children

To evaluate the effect of atropine treatment, we compared annual AL progression rates of the treated Mendelian and non-Mendelian myopes with AL population growth charts of children without treatment using data from the prospective birth cohort study Generation R, in which AL was measured at 6 and 9 years. Percentiles of annual AL elongation (mm/year) were determined separately for boys and girls.^18^

### Mouse model

Our mouse model represented the ocular phenotype of Donnai–Barrow syndrome caused by a mutation in the *LRP2* gene. All mouse experiments were approved by the ethical committee of the Royal Netherlands Academy of Arts and Sciences (KNAW, Amsterdam, the Netherlands), and all treatments and care of mice were performed according to the ARVO Statement for the Use of Animals in Ophthalmic and Vision Research. Mice were socially housed in a contrast-rich environment, under full-spectrum 200 lux illumination, in 18 h light/6 h dark cycle and had access to water and food ad libitum.

The model was a C57BL/6J mouse strain in which *Lrp2* was selectively inactivated in developing ocular structures by means of Foxg1-Cre-mediated inactivation of the gene, as previously described by Cases et al.^19,20^*Foxg1-IRES-CRE* mice were purchased from Jackson Laboratory.^21^*Lrp2*^lox/lox^ mice were imported from Dr Thomas Willnow, Max Delbrück Center for Molecular Medicine, Berlin, Germany.^22^

Male and female mice, homozygous for both *Foxg1*^cre^ and *Lrp2*^lox^ (*Foxg1*^cre/cre^*Lrp2*^lox/lox^) and littermates negative for either *Foxg1*^cre^ or *Lrp2*^lox^ (*Foxg1*^wt/wt^*Lrp2*^lox/lox^, *Foxg1*^cre/cre^*Lrp2*^wt/wt^, *Foxg1*^cre/wt^*Lrp2*^wt/wt^ or *Foxg1*^wt/wt^*Lrp2*^lox/wt^) were included in the study, enabling comparison between eyes with no *Lrp2 knock out* (KO) and eyes with intact *Lrp2* expression (CTRL). We observed that KO mice from breeding with heterozygous parents (breeding pair with genotype *Foxg1*^cre/wt^*Lrp2*^lox/wt^ and/or *Foxg1*^cre/wt^*Lrp2*^lox/lox^) and KO mice from breeding with homozygous parents (with genotype *Foxg1*^cre/cre^*Lrp2*^lox/lox^) differed in ocular biometry. KO mice from heterozygous parents had a significantly larger lens thickness compared with KO mice from homozygous parents in the period P28–P56 (heterozygous vs. homozygous breeding: −30.06 ± 9.29 μm, *p* = 0.002, linear mixed effects [LME] model; Supplement 1). This difference could not be attributed to a difference in body weight (heterozygous vs. homozygous breeding: −0.26 ± 0.73 g, *p* = 0.73, LME model). Other ocular compartments were not significantly different between the breeding pairs (*p* ≥ 0.27, LME model). To avoid heterogeneity in the KO group, we only included KO mice from heterozygous parents for ocular biometry experiments.

### Pedestal surgery in mice

Mice were equipped with a head-mounted pedestal to temporarily fix the head for the administration of atropine, ocular measurements and optokinetic reflex (OKR) recordings. The aluminium pedestal was attached to the parietal bones of the skull using dental cement (Super Bond, sunmedical.co.jp) in a surgical procedure at the age of postnatal day (P)25–P27. Surgery was performed under general anaesthesia (isoflurane with O_2_ and air), the periost was topically anaesthetised using lidocaine (Eurovet Animal Health, eurovet.com.tr) and perioperative meloxicam (2 mg/kg body weight, Metacam, boehringer-ingelheim.com), an analgesic, anti-inflammation and fever drug, was offered by subcutaneous injection.

### Atropine application in mice

From postnatal day 30 to 55, left eyes were treated daily with a droplet of atropine sulphate 1% (Bausch + Lomb Pharma, bausch-lomb.be), while the right eyes were treated with saline. Topical administration was performed in awake head-fixed mice, between 09:00 and 11:00 h.

### Ocular biometry in mice

3D images of the eye were generated using spectral-domain optical coherence tomography (SD-OCT Telesto, thorlabs.com) in anaesthetised mice at P28, P42 and P56. Mice were anaesthetised using ketamine/xylazine (80 and 10 mg/kg body weight, respectively; Aneketin, dechra.us.com; Xylasan, alfasan.nl). Mice were placed into a custom-made mouse holder and aligned with the OCT system in such a way that the pupil plane was approximately perpendicular to the a-scan direction. Using custom MATLAB software, the AL of the eye and the ocular compartments were measured manually from the OCT images (Figure 2a) and converted to geometrical length by dividing the optical length by the average refractive index (RI) of the medium (cornea RI = 1.38, anterior, vitreous and retina RI = 1.34, lens RI = 1.44). The eyes were moistened using artificial tears (Duodrops, ceva.com), while recovering from anaesthesia under an infrared heating lamp. One hour after onset, anaesthesia was reversed using xylazine antagonist atipamezole (1 mg/kg body weight, Antisedan, orion.fi/en/Orion-group/products-and-services/veterinary-drugs/).^23^

### Optokinetic reflex in mice

The OKR was evaluated in untreated KO male mice, and compared with age- and gender-matched wild-type C57BL/6J mice. Mice were placed in a mouse holder, which allowed head-fixation and panoramic vision, and contralateral eyes were covered by a miniature blackout cap for monocular visual stimulation. A panoramic, green monochromatic visual stimulus was created on three high-contrast back-projection screens (1.47 × 1.18 m; Stewart Filmscreen, stewartfilmscreen.com) that were placed around the mouse, using three Optoma DLP projectors (60 fps – optomausa.com). The combined field of view was 270° × 77.5°. The visual stimulus for the OKR consisted of sine wave gratings ranging from 0.03 to 0.60 cycles/degree, moving at a fixed speed of 10 degrees/s. The average luminance was 15 cd/m^2^ and average Mickelson contrast was 92%. Stimuli were generated by a computer and corrected for perspective distortion by projection onto a virtual cylinder centred on the animal's head. Eye movements were recorded using an infrared video tracking system (JAI RM-6740CL monochrome charged-couple device (CCD) camera, 200 fps, jai.com). 2D eye position was computed from the relative distance between pupil centre, corneal reflections of the infrared LEDs^24,25^ and pupil size.^26^ Epochs containing saccades, eye blinks and motion artefacts were excluded from analysis. Eye velocity was smoothed using a Gaussian smoothing kernel with a SD of 7.5 ms (25-Hz cutoff). The gain was calculated as the average eye velocity divided by the average stimulus velocity and visualised for each spatial frequency.

### Dopamine and DOPAC measurement

Levels of dopamine (DA) and its metabolite 3,4-dihydroxyphenylacetic acid (DOPAC) in the retina of atropine- and saline-treated eyes were determined by high-performance liquid chromatography (HPLC), 2 and 24 h after atropine treatment.

Mice were deeply anaesthetised using general isoflurane/O_2_ and sacrificed by cervical dislocation. Retinal tissue was immediately isolated, put on dry ice and kept at −80°C until further processing. DA and DOPAC retinal content exhibit a circadian rhythmicity.^27^ Therefore, we isolated all retinas between 14:00 and 16:00 h. To minimise variability, interocular comparisons were performed.

Retinas were homogenised in 100 μL cold sonication fluid (0.01% w/v cysteine, 0.1 N perchloric acid, 130 nM 3MT [a-Methylserotonin-maleate] as an internal standard) via sonication for 5–7 s. After homogenizing, samples were spun in a centrifuge to pellet cell organelles. An 80 μL aliquot of supernatant was collected in an Eppendorf vial, and 4 μL of cold 7.7 N KOH was added to precipitate PCA ions. The solution was stirred for 30 s, put onto melting ice for at least 10 min and subsequently stirred again for 15 s and centrifuged at 17.2 *g* for 10 min at 4°C. Supernatant (5 μL) was injected per ALF-115 column (150 mm × 1 mm, 3 μm C18) of the HPLC ALEXYS 100 2D system equipped with electrochemical detection (DECADE II; antecscientific.com). The mobile phase for the ALF-115 columns consisted of 100 mM phosphoric acid, 10 mM KCl, 2.43 mM octanesulphonic acid (OSA), 13.5 μM EDTA and 16% methanol in milliQ water, with an adjusted pH of 3.36. The flow rate was kept constant at 40 μL/min. Separation was performed at 38°C and the electrochemical potential were set at 670 mV against an in-situ Ag/AgCl (ISAAC) electrochemical cell. Signals were analysed using Clarity software (dataapex.com). Samples were normalised to total retina mass and for stability during the overnight HPLC-run.

### Statistical analysis

In the children study, the primary outcome was the annual progression rate of AL (mm/year). Statistically significant differences in baseline AL were calculated with a two-sample *t*-test; annual AL progression rate and their predicted AL progression rates without atropine were tested using one-way ANOVA. We calculated the difference in AL progression between the treated groups and stratified this for inherited retinal dystrophies or other ocular-limited disorders and connective tissue disease. Statistical differences were evaluated using the Kruskal–Wallis test.

In the mouse study, the study outcomes were ocular biometry and OKR gain. Differences in outcomes between genotypes were tested using LME models. In these models, we controlled for genotype, gender and age as fixed factors, for correlation between eyes using mouse ID as random effect factor. Differences in AL between atropine- and saline-treated eyes and potential interaction with genotype and gender were tested using a three-way ANOVA. Differences between baseline AL and AL measured at 4 h after the eye drop was instilled were tested for atropine- and saline-treated eyes using two-sample *t*-tests. Differences in dopamine and DOPAC between atropine- and saline-treated eyes and potential interaction with genotype and gender were also tested using a three-way ANOVA.

All data are presented as mean value ± standard error of the mean (SEM) unless indicated otherwise. Statistically significant *p-*values were indicated by asterisks (*p = 0.01–0.05; **p = 0.001–0.01; ***p = 0.0001–0.001; and ****p = 0.0001). All statistical tests were performed using MATLAB (R2016B, mathworks.com).

## RESULTS

Our group of Mendelian myopes consisted of 14 children with, at baseline, a median age of 6 years (range: 2–13 years, IQR: 3.8), a median AL of 25.9 mm (range: 23.8–28.6 mm; IQR: 1.2) and a median SE of −7.5 D (range: −9.8 to 5.0 D, IQR: 4.8; Table 1). Eight children were boys. Twelve children were treated with 0.5% atropine; two children received 1% atropine.

**TABLE 1:** Characteristics at baseline of study populations.

	Mendelian myopic children (*n* = 14)	Non-Mendelian myopic children (*n* = 36)	*p*-Value
Age			
≤4 years	6	8	0.21
5 to 7 years	4	12	
8 to 10 years	2	12	
≥11 years	2	4	
Median age (range, IQR) in years	6 (2 to 13, 3.8)	7 (3 to 13, 3.3)	
Gender			
Male	8	26	0.32
AL			
≤24 mm	2	2	0.52
24 to 26 mm	5	23	
26 to 28 mm	6	10	
≥28 mm	1	1	
Median AL (range, IQR) in mm	25.9 (23.8 to 28.6, 1.2)	25.9 (23.8 to 28.1, 1.4)	
Spherical equivalent (SE)			
≤−10 D	4	9	0.74
−10 to 8 D	2	6	
−8 to −6 D	3	7	
≥−6 D	5	14	
Median SE (range, IQR) in D	−7.5 (−11.4 to 4.4, 4.8)	−7.0 (−12.1 to 2.6, 5.0)	

Abbreviations: AL, axial length; D, dioptre; IQR, interquartile range.

Matching of Mendelian versus non-Mendelian myopes is presented in Supplement 2. In total, 36 non-Mendelian myopes were matched; on average, three matches for each Mendelian myope (range: 1–7 matches, IQR: 1). All these children received atropine 0.5%.

At baseline, non-Mendelian myopes had a median age of 7 years (range: 3–13 years, IQR: 3.3), a median AL of 25.9 mm (range: 23.8–28.1 mm, IQR: 1.4) and a median SE of −7.0 D (range: −12.1 to 2.6 D, IQR: 5.0) (Wilcoxon rank-sum test, Table 1). All non-Mendelian myopes received 0.5% atropine; 26 of them were boys. The majority of the non-Mendelian myopes had parents without high myopia (22/36). Six children had one parent with high myopia and one child had both parents with high myopia.

For each Mendelian myope, AL at baseline was plotted against the average of matched, non-Mendelian myopes (Supplement 3).

**FIGURE 1:**
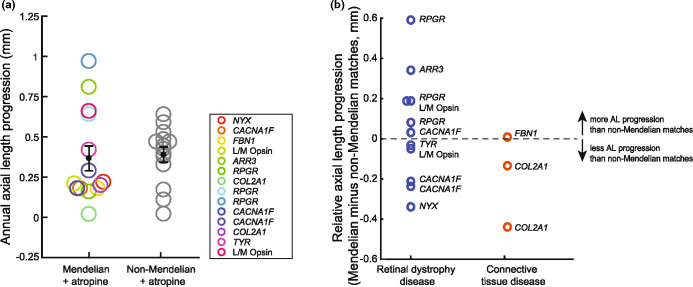
Response to high-dose atropine in Mendelian myopes and the matched non-Mendelian myopes. (a) Annual axial length (AL) progression of Mendelian and non-Mendelian myopes during high-dose atropine treatment. Mendelian and non-Mendelian myopes did not have a significantly different AL progression rate (*p* = 0.80, two-sample *t*-test). (b) Relative to non-Mendelian myopia, mutations in retinal dystrophy genes corresponded with +0.05 ± 0.08 mm more AL progression; mutations in connective tissue genes with −0.19 ± 0.13 mm less progression during atropine treatment (*p* = 0.17, two-sample *t*-test).

For the Mendelian group, annual AL progression during atropine treatment was on average +0.37 ± 0.08 mm (Figure 1a). For the matched non-Mendelian myopes, annual AL progression was +0.39 ± 0.05 mm (*p* = 0.80, two-sample *t*-test).

To evaluate the effect of atropine on AL growth reduction, we plotted the AL growth rate for the Mendelian and non-Mendelian children in the AL growth chart of untreated children from Generation R (Supplement 4).^18^ Although the majority (71%) of the Mendelian and non-Mendelian children in this study had ALs above the 99th percentile of the growth chart, we used this percentile as a comparison. The 99th percentile represented 0.55 mm/year (boys) and 0.50 mm/year (girls). A small proportion of children (29%) had AL between the 93 and 98 percentile, which corresponded with progression rates between 0.24–0.44 mm/year. From all these percentiles, we estimated an expected annual AL progression for uncontrolled myopia of 0.47 ± 0.03 mm/year (Supplement 5) and calculated a growth reduction of 27% (+0.37 mm/year) and 23% (+0.39 mm/year) for Mendelian and non-Mendelian myopia, respectively. The small samples sizes hampered achieving statistical significance for these differences (*p* = 0.36 and *p* = 0.53, respectively, ANOVA, multiple comparisons).

Children diagnosed with mutations in retinal dystrophy genes (*n* = 10; *CACNA1F*, *NYX*, *L/M opsin*, *RPGR*, *TYR*) had on average +0.05 ± 0.08 mm more AL progression relative to their non-Mendelian matches (Figure 1b); while children with mutations in connective tissue genes (*n* = 3; *FBN1*, *COL2A1*) had on average − 0.19 ± 0.13 mm less AL progression. Again, these differences failed to achieve statistical significance (*p* = 0.17, two-sample *t*-test).

### Ocular phenotype of *Lrp2* KO mice

**FIGURE 2:**
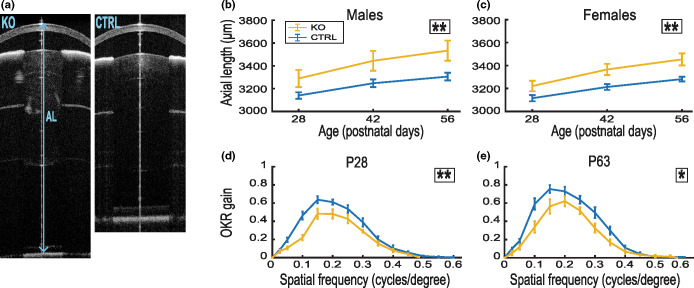
Phenotypic characterisation of the eyes of Foxg1^cre/cre^Lrp2^lox/lox^ (KO) mice, compared with control (CTRL) littermates. (a) spectral-domain ocular coherence tomography (SD-OCT) images of (left) knock out (KO) and (right) CTRL eyes. The blue arrow indicates the axial length (AL). (b, c) Change in AL over the course of 1 month from P28 in male and female mice. Ocular AL in KO mice was significantly larger compared with CTRL littermates in the period P28–P56 for both sexes. (d, e) OKR gain to horizontally moving (10 degrees/s) vertical sinusoidal grating patterns. In mice, the maximally resolvable spatial resolution is about 0.5 cycles per degree (cpd). At 10 dps image velocity, OKR gain in mice typically peaks between 0.1 and 0.2 cpd. OKR over the entire spatial frequency range was significantly reduced in KO mice, compared with gender- and age-matched CTRLs, at both P28 (d) and P63 (e). Group sizes: AL: males, KO *n* = 7, CTRL *n* = 9; females, KO *n* = 6, CTRL *n* = 8; OKR: only males, KO *n* = 7, CTRL *n* = 8. OKR, optokinetic reflex. Statistically significant differences between genotypes were presented in the upper right corner of the graphs. *p = 0.01–0.05; **p = 0.001–0.01; linear mixed-effects model. P, days post natal.

The eyes of *Lrp2* KO mice had a significantly larger AL compared with the eyes of littermate CTRL mice (P28: male, KO: 3289.2 ± 75.1, CTRL: 3140.0 ± 28.4; female, KO: 3221.2 ± 45.7, CTRL: 3115.2 ± 27.3; P56: male, KO: 3533.5 ± 88.7, CTRL: 3305.6 ± 33.2; female, KO: 3453.4 ± 52.3, CTRL: 3281.7 ± 20.4 μm; *p* = 0.002, LME model; Figure 2a–c). The ocular elongation in the *Lrp2* KO could be fully attributed to a significantly elongated vitreous chamber (P28: male, KO: 895.6 ± 95.3, CTRL: 718.0 ± 12.4; female, KO: 839.1 ± 59.5, CTRL: 706.5 ± 14.9; P56: male, KO: 870.7 ± 105.0, CTRL: 638.3 ± 6.7; female, KO: 775.9 ± 68.5, CTRL: 627.1 ± 6.6 μm; *p* = 0.002, LME model; Supplement 6). No significant differences were found between the genotype groups regarding body weight, corneal thickness, lens thickness, anterior chamber depth and retinal thickness (Supplement 6).

KO mice had a significantly reduced OKR gain compared with gender- and age-matched CTRL mice (P28, *p* = 0.004; P63, *p* = 0.04, LME model; Figure 2d,e).

### Atropine treatment in mice

At the start of the atropine treatment, no significant differences in ocular biometry values were observed between the right and left eyes in any of the groups (all *p* ≥ 0.001, two-sample *t*-test).

**FIGURE 3:**
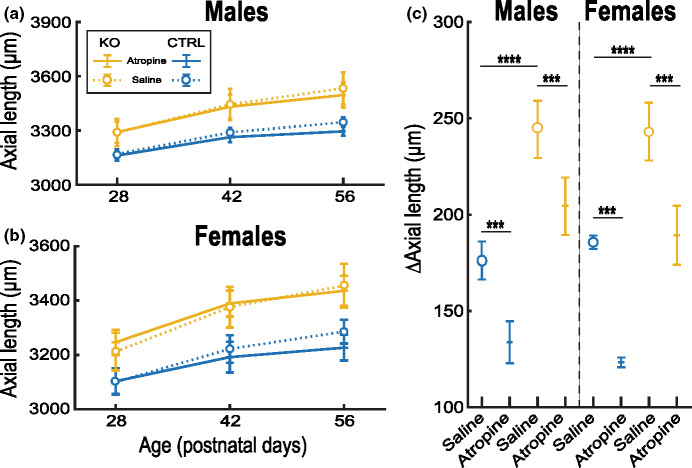
Effects of daily topical application of 1% atropine on the axial length (AL) of mice featuring the ocular phenotype of Donnai–Barrow syndrome (Foxg1^cre/cre^Lrp2^lox/lox^, knock out (KO)) and control (CTRL) littermates. (a, b) Comparison between saline-treated and atropine-treated eyes regarding the change in AL over the course of 1 month from P28 in male (a) and female (b) mice. (c) Comparison between saline-treated and atropine-treated eyes regarding the change in AL over the course of 1 month. AL in KO mice was significantly longer at the start of the treatment (a, b) and increased more during the treatment (P28–P56) compared with CTRL mice. The net change in AL was significantly reduced in atropine-treated eyes compared with saline-treated eyes in both KO and CTRL mice. AL reduction by atropine was not significantly different between KO and CTRL eyes, nor between male and female mice. Group sizes: male, KO *n* = 18, CTRL *n* = 14; female, KO *n* = 14, CTRL *n* = 8; ***p = 0.0001–0.001, ****p = 0.00001; ANOVA. P, days post-natal.

In the saline-treated eyes, AL growth was significantly larger in KO mice compared with CTRL littermates in the period P28–P56 (∆AL: male, KO: 244.3 ± 14.9, CTRL: 176.1 ± 9.9; female, KO: 242.7 ± 14.6, CTRL: 185.4 ± 3.2 μm; *p* < 0.001; ANOVA; Figure 3). In this period, lens thickness increased at the expense of the vitreous chamber, reducing it in size. This reduction was significantly smaller in KO mice than in CTRL mice (∆VCD: male, KO: −25.0 ± 16.7, CTRL: −91.6 ± 5.8; female, KO: −56.3 ± 17.5, CTRL: −96.0 ± 14.1 μm; *p* < 0.001, ANOVA, Supplement 7).

Atropine significantly reduced AL growth in both KO and CTRL mice, in both males and females (∆AL: atropine minus saline: male, KO: −39.7 ± 10.7, CTRL: −42.4 ± 6.6, female, KO: −53.4 ± 1.4, CTRL: −62.1 ± 4.0 μm; treatment effect: *p* < 0.001, ANOVA, Figure 3). The proportion of AL reduction by atropine was not significantly different between KO and CTRL eyes, nor between male and female mice (interaction genotype x treatment *p* = 0.2; interaction gender × treatment *p* = 0.6, three-way ANOVA). Atropine reduced AL growth by 28% in the male and 27% in the female KO mice. The atropine-induced reduction was mostly due to reduced ACD growth (∆ACD: atropine minus saline: male, KO: −53.5 ± 6.1, CTRL: −42.4 ± 5.5; female, KO: −50.4 ± 2.6, CTRL: −46.8 ± 2.7 μm; *p* < 0.001, ANOVA, Supplement 7).

To check whether the effect of atropine was immediate, we measured ocular biometry prior to and ±4 h after the first application and compared this with the saline-treated eyes. No significant effects of atropine were observed in any of the ocular biometric measures for any of the groups (relative change atropine – relative change saline: AL: male, KO: −0.1 ± 0.4, CTRL: 0.2 ± 0.3; female, KO: −0.30 ± 1.0, CTRL: −0.30 ± 0.3%; all *p* > 0.05; two-sample *t*-test; Supplement 8).

### Retinal dopamine and DOPAC levels in KO and CTRL mice treated with atropine

**FIGURE 4:**
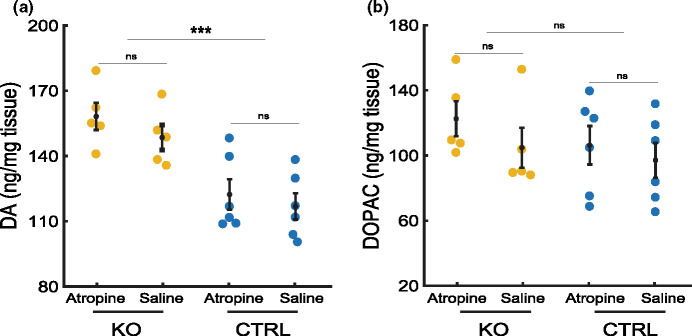
Dopamine (DA) and 3,4-dihydroxyphenylacetic acid (DOPAC) retinal levels of mice featuring the ocular phenotype of Donnai–Barrow syndrome (Foxg1^cre/cre^Lrp2^lox/lox^, knockout (KO)) and control (CTRL) littermates with eyes treated with 1% atropine and compared with saline. (a) DA levels showed slight, nonsignificant increases in atropine-treated eyes, compared with saline-treated eyes. DA levels were significantly increased in KO mice compared with CTRL littermates. Effect of gender and interactions between treatment, genotype or gender were not statistically significant on DA levels. (b) Effects of DOPAC levels were not significantly changed by atropine treatment, genotype or gender. Group sizes: KO *n* = 5, CTRL *n* = 6 mice; ***p = 0.0001–0.001; three-way ANOVA.

Effects of atropine, *Lrp2* KO and gender on DA and DOPAC levels were examined using a three-way ANOVA. KO mice had significantly increased DA levels compared with CTRL littermates (saline, KO: 148.5 ± 5.8, CTRL: 116.9 ± 6.0, main effect genotype *p* < 0.001, Figure 4a, Supplement 9); but DOPAC level and DOPAC/DA ratio were not significantly different (DOPAC: saline, KO: 104.8 ± 12.3, CTRL: 97.1 ± 10.8, main effect genotype *p* = 0.41; DOPAC/DA: saline, KO: 0.71 ± 0.1, CTRL: 0.8 ± 0.1, main effect genotype *p* = 0.21, Figure 4b, Supplement 9). Gender had no significant effect on DA, DOPAC levels or DOPAC/DA ratio (ANOVA tables in Supplement 9).

In both KO and CTRL mice, atropine-treated eyes showed slightly increased levels of DA and DOPAC 2 h after a single application compared with saline-treated eyes, but this difference did not reach statistical significance (DA: KO, atropine: 158.2 ± 6.3, saline: 148.5 ± 5.8; CTRL, atropine: 122.3 ± 7.0, saline: 116.9 ± 6.0, simple main effect treatment *p* = 0.29; DOPAC: KO, atropine: 122.5 ± 10.8, saline: 104.8 ± 12.3; CTRL, atropine: 106.2 ± 11.8, S: 97.1 ± 10.8, simple main effect treatment *p* = 0.27; Figure 4). Ratio DOPAC/DA, commonly used as an indicator for DA turnover,^28,29^ was not significantly different between the groups (KO, atropine: 0.8 ± 0.1, saline: 0.7 ± 0.1; CTRL, atropine: 0.9 ± 0.1, S: 0.8 ± 0.1, simple main effect treatment *p* = 0.29, Supplement 9). Likewise, we did not find any statistically significant interactions between the effects of treatment, genotype or gender for levels of DA, DOPAC nor the ratio of DOPAC/DA (ANOVA tables in Supplement 9).

In an additional experiment, we determined DA and DOPAC levels 24 h after the final application of daily atropine over 4 weeks. No statistically significant effects of treatment on the levels of DA, DOPAC or the DOPAC/DA ratio were observed (Supplement 10). Interestingly, KO mice showed significantly increased DOPAC/DA ratios compared with CTRL littermates (KO, atropine: 0.3 ± 0.0, saline: 0.4 ± 0.0; CTRL, atropine: 0.3 ± 0.0, saline: 0.2 ± 0.0, simple main effect genotype *p* = 0.04, Supplement 10). A significant interaction between genotype and gender was observed for the DOPAC/DA ratio, *F*(1, 36) = 5; *p* = 0.03.

## DISCUSSION

This study aimed to explore the effectiveness of atropine for Mendelian forms of high myopia in children as well as in a mouse model. High-dose atropine treatment in highly myopic children diagnosed with a monogenic syndrome achieved a similar reduction of AL as in children with a comparable non-Mendelian myopia phenotype. Although progression rates during treatment were high in both groups (0.37 and 0.39 mm/year, respectively), they appeared to be lower than the rates estimated from charts of uncontrolled eye growth (0.47 mm/year). This assumption was validated in the mouse model. In mice with an eye-specific KO of the *Lrp2* gene responsible for the high-myopia Donnai–Barrow syndrome, we observed extremely elongated eyes, confirming the previous findings of Cases et al.^19,20^ Atropine reduced AL elongation by 28%. Strikingly, the DA level and DOPAC/DA ratio were significantly increased in *Lrp2* KO mice compared with littermates CTRL mice. Atropine raised DA and DOPAC levels somewhat further, but this increase was not significant.

Atropine has become an accepted treatment for progressive myopia, and a high dose (0.5%; 1%) is often preferred in children with very long ALs and fast rates of progression.^12^ In children with moderate myopia, this dosage is known to limit the growth rate in the first year up to 0.05 mm/year,^17^ but whether it would also work for Mendelian disorders with very high degrees of myopia has been a matter of debate. Nevertheless, previous studies have provided evidence for a need for myopia control in at least some of these disorders. For example, the retinal degenerative changes and deteriorating retinal function in CSNB appear to be due mostly to complications of high myopia, not to the night blindness.^10^ In retinitis pigmentosa due to mutations in *RPGR*, patients with higher levels of myopia have a faster decline in visual acuity.^9^ Based on findings from the general population, we know that each dioptre matters; a reduction of 1 D myopia lowers the risk of myopic retinopathy, the most common and serious sight-threatening complication of myopia, by 40%.^30-34^ Taken together, it seems rational to also reduce the severity of myopia in Mendelian disorders to prolong the period of functional vision.

In our study, children with high myopia due to a Mendelian disorder had the same response to high-dose atropine treatment as children with high levels of myopia without accompanying pathology. Nevertheless, eye growth was still considerable and one could question whether atropine had any effect at all. We did not consider non-treatment or treatments with less efficacy an ethical option for these fast progressive myopes, and archives of well-documented uncontrolled eye growth in young, highly myopic children from the clinic are rare. We therefore turned to our population-based birth-cohort Generation R,^18^ and calculated an average annual AL progression of 0.47 mm/year for untreated children on the percentile closest to the baseline AL and age in children from this study. Using this reference for uncontrolled growth, atropine appeared to reduce AL progression by 27% in children with a strong monogenic driver for myopia.

Although limited by the small sample size, we attempted to evaluate differences in treatment response by type of Mendelian disorder. We noticed that AL elongation, particularly in retinitis pigmentosa due to mutations in *RPGR*, was difficult to control (Figure 1b). By contrast, the growth rate in CSNB and Stickler syndrome seemed to respond better to high-dose atropine. As expected, differences did not reach statistical significance, and larger study samples are needed to provide more profound evidence for this impression.

The mouse model provided experimental proof for reduction of AL elongation by atropine in Mendelian myopia. Contrary to the human eye, the mouse eye has a very small vitreous chamber, a wide anterior chamber and a large and rigid lens.^35^ This difference in ocular biometry between the species might explain why atropine predominantly affected the growth of the anterior chamber in mice, whereas it mostly affects the growth of the vitreous chamber in humans.^36,37^ To rule out a change in dimensions due to the cycloplegic effect of atropine, we measured ocular biometry shortly after the application of the drug. We did not observe any immediate changes, indicating that the measured changes over time were true differences in AL growth. Whether our observed findings in mice in which *Lrp2* had been selectively knocked out may be generalised to atropine treatment in all Mendelian myopia is uncertain. Similar to our results in children, atropine in mice may be a more effective inhibitor of eye growth for some genetic drivers than others. This reservation notwithstanding, dysfunction of *Lrp2* in mice and *LRP2* in humans is one of the strongest known single genetic drivers of myopia, and AL over 30 mm before 10 years of age in patients with Donnai–Barrow is not uncommon (JRP and CCWK clinical observation). If atropine manages to counteract eye growth in this gene model, then it is likely to be beneficial when genetic effects are less robust.

The mechanism of action of atropine, a nonselective muscarinic acetylcholine receptor antagonist, is still unresolved.^11^ A common hypothesis assumes that modulation of DA retinal signalling underlies atropine's protective effect. In chickens, a reduction of experimentally-induced myopia has been shown to coincide with increased retinal and vitreal levels of DA.^15,16^ However, a recent pharmacological study, also performed in chickens, opposed this hypothesis by demonstrating that neither the D2-like receptor antagonist Spiperone nor the D1-like receptor antagonist SCH-23390 was able to block the protective effects of atropine.^38^ Their experiments also showed that three different concentrations of atropine protected against experimentally induced myopia; only the highest concentration induced increased levels of DA and DOPAC. An in vitro study provided evidence that atropine appears to block myopia via α2-adrenoceptors rather than muscarinic receptors;^39^ Thomson et al. also reported that several agents with cholinergic agonistic capacity can inhibit myopic growth.^38^ Taken together, these results suggest that atropine does not exert its effect through cholinergic receptors, and DA is not its major mediator. Our findings in mice are in line with this notion. We showed that the anti-myopic effect was not accompanied by significantly increased DA and DOPAC levels after atropine application. Unexpectedly, the DA level and its turnover rate were significantly increased in our myopia mouse model of Donnai–Barrow syndrome. To our knowledge, DA is not involved in this syndrome. Whether the elevated levels are a consequence of the high-myopia phenotype with subsequent lower contrast sensitivity is unclear, and this intriguing finding in high myopia needs further exploration.

In summary, this study found evidence for myopia control in Mendelian forms of high myopia in children and mice. The similarity in results in both these species strongly suggests that atropine can have merit in counteracting fast progressive myopia in monogenic ocular syndromes. Our findings are in need of validation in larger studies with many patients of a diverse Mendelian background to identify which ophthalmogenetic disorders with high myopia benefit most from atropine treatment.

## Supplementary Information

Supplementary file (DOCX 1.09 MB)
