# Supplementary material for: Myopia control in Mendelian forms of myopia
Source: Ophthalmic Physiol Opt. 2023 Mar 7;43(3):494–504. doi: 10.1111/opo.13115 (PMC12852259; doi:10.1111/opo.13115)
Supplement: Supplementary file 1 — Supplementary file (DOCX 1.09 MB) [file 44402_2023_4303021_MOESM1_ESM.docx]

**Supplementary Information**

**Suppl. I:** Comparison of ocular biometry and body weight between *Foxg1*^cre/cre^*Lrp2*^lox/lox^ mice bred from heterozygous breeding and from homozygous breeding.

**Suppl. 2:** Table of characteristics of the children with a Mendelian form of myopia and the matched non-Mendelian myopes.

**Suppl. 3:** Axial length at baseline for Mendelian myopes, and the average of the matched non-Mendelian myopes.

**Suppl. 4:** Annual axial length growth rates of the myopic Mendelian and non-Mendelian children during high dose atropine treatment plotted in growth charts from Generation R.

**Suppl. 5:** Table of annual axial length progression during high dose atropine treatment in children with Mendelian myopia, the average of the matched non-Mendelian myopes, compared to their expected progression without treatment.

**Suppl. 6:** Comparison of ocular biometry and body weight between *Foxg1*^cre/cre^*Lrp2*^lox/lox^ and control mice.

**Suppl. 7:** Effect of atropine on vitreous chamber depth and anterior chamber depth in *Foxg1*^cre/cre^*Lrp2*^lox/lox^ and control mice.

**Suppl. 8:** Immediate effect of atropine on ocular biometry, relative to saline, in *Foxg1*^cre/cre^*Lrp2*^lox/lox^ and control male and female mice.

**Suppl. 9:** Dopamine, 3,4-dihydroxyphenylacetic acid retinal levels and the ratio between them of *Foxg1*^cre/cre^*Lrp2*^lox/lox^ and control mice, two hours after a single application of atropine, and compared with saline (figure and ANOVA table).

**Suppl. 10:** Dopamine, 3,4-dihydroxyphenylacetic acid retinal levels and the ratio between them of *Foxg1*^cre/cre^*Lrp2*^lox/lox^ and control mice, 24 hours after final atropine application of 4 weeks daily and compared with saline (figure and ANOVA table).


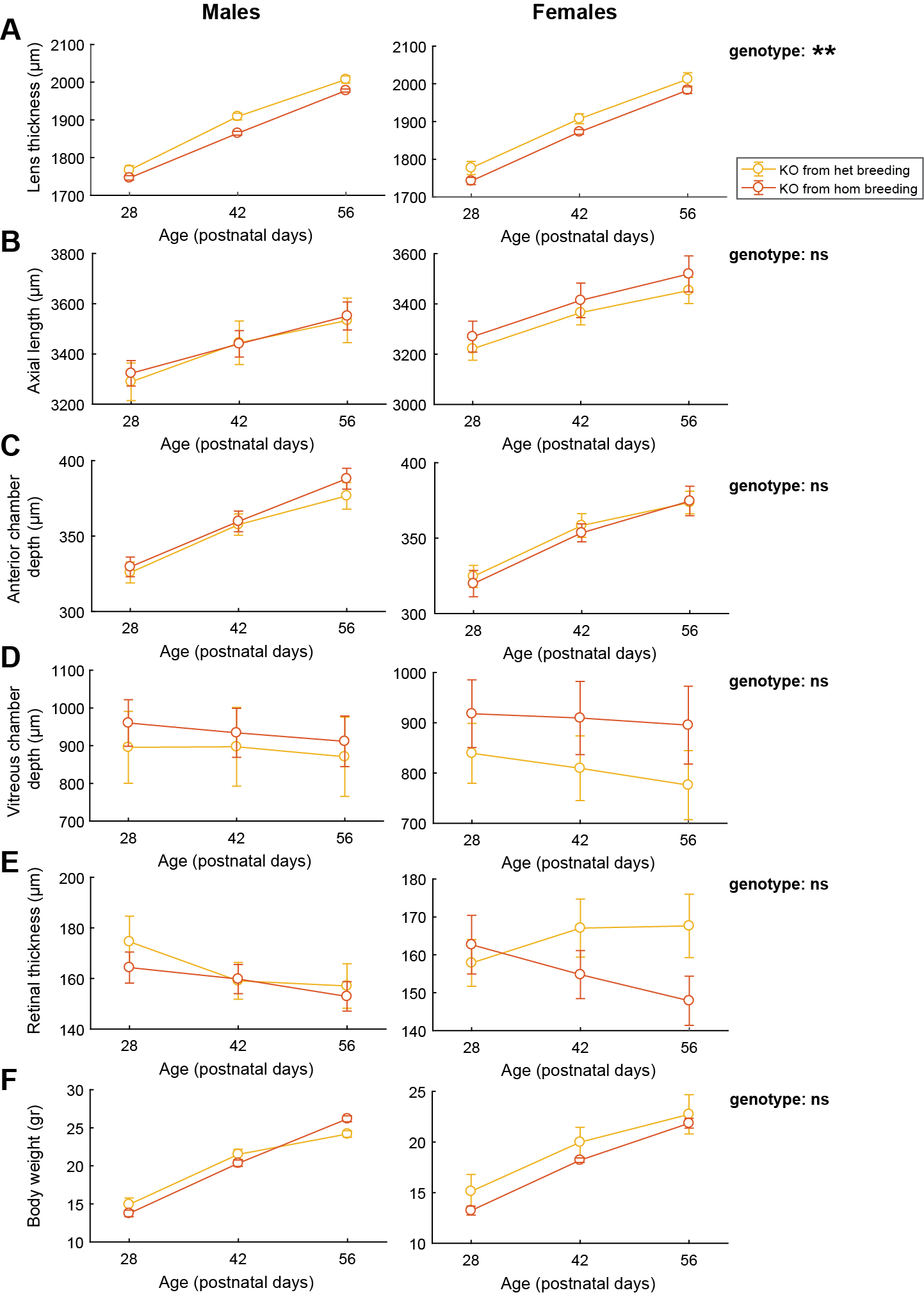


***Suppl. 1: Comparison of ocular biometry and body weight between Foxg1^cre/cre^Lrp2^lox/lox^ (KO) mice bred from heterozygous (het.) breeding (Foxg1^cre/wt^Lrp2^lox/wt^ and/or Foxg1^cre/wt^Lrp2^lox/lox^) and Lrp2 KO mice from homozygous (hom.) breeding (Foxg1^cre/cre^Lrp2^lox/lox^ ).*** *(****A****). KO mice from het. breeding showed statistically significant greater lens thickness compared to KO mice from hom. breeding. (****B****) total axial length, (****C****) anterior chamber depth, (****D****) vitreous chamber depth, (****E****) retinal thickness, and (****F****) body weight did not show statistically significant differences between the breeding strategies. Group sizes: male, het. breeding n=7, hom breeding n=16; female het. breeding n=6, hom. breeding n=7 eyes; ns, not significant; ∗∗ p < 0.001–0.01. Linear mixed effects model.*

**Supplementary 2**

|  | **#8**  **Mendelian** | **#8**  **average non-Mendelian** | **#9**  **Mendelian** | **#9**  **average non-Mendelian** | **#10**  **Mendelian** | **#10**  **average non-Mendelian** | **#11**  **Mendelian** | **#11**  **average non-Mendelian** | **#12**  **Mendelian** | **#12**  **average non-Mendelian** | **#13**  **Mendelian** | **#13**  **average non-Mendelian** | **#14**  **Mendelian** | **#14**  **average non-Mendelian** |
| --- | --- | --- | --- | --- | --- | --- | --- | --- | --- | --- | --- | --- | --- | --- |
| **Number of non-mendelian matches** | - | 1 | - | 1 | - | 2 | - | 2 | - | 1 | - | 3 | - | 2 |
| **Gender (0=boy; 1=girl)** | 1 | 1 | 1 | 0 | 0 | 0 | 0 | 0 | 1 | 0 | 0 | 0 | 0 | 0 |
| **Age at baseline (yrs)** | 3 | 4 | 2 | 3 | 4 | 4 | 5 | 6 | 7 | 8 | 6 | 6.33 | 4 | 3 |
| **AL at baseline (mm)** | 25.49 | 25.59 | 23.92 | 23.76 | 23.81 | 24 | 27.32 | 26.26 | 28.58 | 28.06 | 26.37 | 26.15 | 26.32 | 26.03 |
| **Atropine dose (1=1%; 2=0.5%)** | 2 | 2 | 2 | 2 | 1 | 2 | 1 | 2 | 2 | 2 | 2 | 2 | 2 | 2 |
| **Diagnosis Mendelian myopia** | RPGR X-linked carrier | - | RPGR X-linked carrier | - | CSNB (CACNA1F) | - | CSNB (CACNA1F) | - | Stickler | - | Oculocutaneous albinism | - | Bornholm eye disease | - |

|  | **#1**  **Mendelian** | **#1**  **average non-Mendelian** | **#2**  **Mendelian** | **#2**  **average non-Mendelian** | **#3**  **Mendelian** | **#3**  **average non-Mendelian** | **#4**  **Mendelian** | **#4**  **average non-Mendelian** | **#5**  **Mendelian** | **#5**  **average non-Mendelian** | **#6**  **Mendelian** | **#6**  **average non-Mendelian** | **#7**  **Mendelian** | **#7**  **average non-Mendelian** |
| --- | --- | --- | --- | --- | --- | --- | --- | --- | --- | --- | --- | --- | --- | --- |
| **Number of non-mendelian matches** | - | 1 | - | 5 | - | 2 | - | 5 | - | 2 | - | 2 | - | 7 |
| **Gender (0=boy; 1=girl)** | 0 | 0 | 0 | 0 | 1 | 1 | 0 | 0 | 1 | 0 | 0 | 0 | 1 | 1 |
| **Age at baseline (yrs)** | 4 | 3 | 8 | 8.4 | 13 | 12 | 7 | 6.8 | 2 | 3 | 12 | 12.5 | 8 | 8.6 |
| **AL at baseline (mm)** | 25.51 | 25.87 | 26.35 | 26.17 | 24.67 | 24.51 | 26.06 | 26.02 | 26.09 | 26.03 | 25.69 | 25.51 | 24.98 | 24.77 |
| **Atropine dose (1=1%; 2=0.5%)** | 2 | 2 | 2 | 2 | 2 | 2 | 2 | 2 | 2 | 2 | 2 | 2 | 2 | 2 |
| **Diagnosis Mendelian myopia** | CSNB (NYX) | - | CSNB (CACNA1F) | - | Marfan | - | Bornholm eye disease | - | Myopia-26 (ARR3) | - | RPGR mutation | - | Stickler | - |

***Suppl. 2: Characteristics of the children with a Mendelian form of myopia and the matched non-Mendelian myopes.*** *Mendelian and non-Mendelian myopes were matched based on baseline axial length (AL; maximum 2 mm range), age (max 1 year of range), and high dose atropine (0.5-1%). Number of non-Mendelian matches, gender, age at baseline, AL at baseline, atropine dose, Annual AL progression rate, and diagnosis of Mendelian myopia were described.*

**Supplementary 3**

**
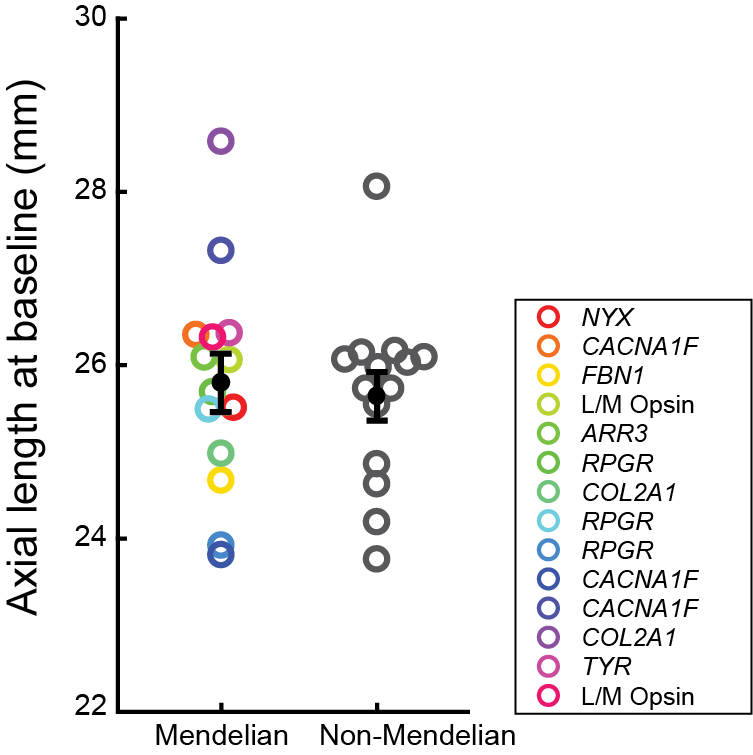
**

***Suppl. 3: Axial length at baseline for Mendelian myopes, and the average of non-Mendelian myopes who were matched based on baseline axial length, age and atropine dose.*** *Axial length at baseline was not significantly different between those groups (p=0.80, n=14, two-sample t-test).*

**Supplementary 4**


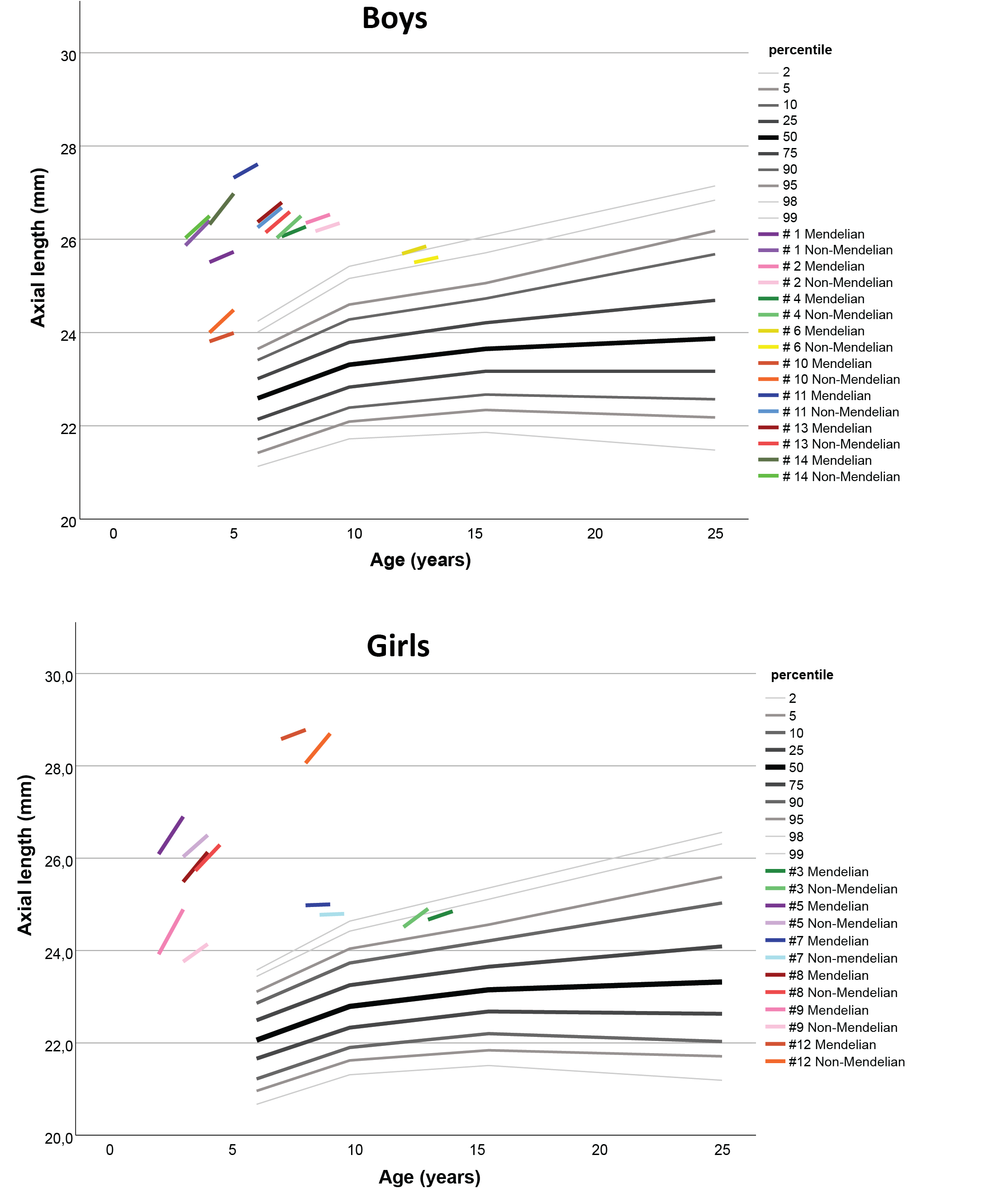


***Suppl. 4: Annual axial length growth rates of the myopic Mendelian and non-Mendelian children during high dose atropine treatment (coloured lines) plotted in growth charts of boys and girls who were not treated from Generation R (adopted from Tideman et al, 2018).*** *Ten out of the 14 children with Mendelian myopia had an axial length above the 99^th^ percentile of the growth chart; four children had an axial length between 93-97 percentiles.*

**Supplementary 5**

***Suppl. 5: Annual axial length (AL) progression from baseline to 1 year during high dose atropine treatment in children with Mendelian myopia, the average of the matched non-Mendelian myopes, compared to their expected annual AL progression without treatment.*** *For Mendelian and non-Mendelian myopes, AL growth percentiles were determined based on gender, baseline AL and age, presented in Tideman et al, 2018. Ten out of the 14 children had an AL above the 99^th^ percentile; four out of the 14 had an AL between 93-98 percentile. Based on these percentiles, annual AL progression rate was estimated for each Mendelian myope and non-Mendelian match. This expected annual AL progression rate without treatment was compared to the measured progression rates of the myopes in order to estimate the effect of atropine. Bottom row represents mean and standard error of the mean (SEM) of the annual AL progression rates.*

| **#** | **Mendelian children** | | | | **Non-Mendelian children** | | | | **Growth chart of untreated children from Generation R** | |
| --- | --- | --- | --- | --- | --- | --- | --- | --- | --- | --- |
|  | **Gender (0=boy; 1=girl)** | **Age at baseline (yrs)** | **AL at baseline (mm)** | **Annual AL progression, baseline to 1 year (mm/yr)** | **Gender (0=boy; 1=girl)** | **Average age at baseline (yrs)** | **Average AL at baseline (mm)** | **Average annual AL progression, baseline to 1 year (mm/yr)** | **Percentile AL** | **Expected annual AL progression, baseline to 1 year (mm/yr)** |
| 1 | 0 | 4 | 25.51 | 0.22 | 0 | 3 | 25.87 | 0.53 | >99 | 0.55 |
| 2 | 0 | 8 | 26.35 | 0.18 | 0 | 8.4 | 26.17 | 0.17 | >99 | 0.55 |
| 3 | 1 | 13 | 24.67 | 0.18 | 1 | 12 | 24.51 | 0.40 | 93 | 0.24 |
| 4 | 0 | 7 | 26.06 | 0.21 | 0 | 6.8 | 26.02 | 0.33 | >99 | 0.55 |
| 5 | 1 | 2 | 26.09 | 0.81 | 0 | 3 | 26.03 | 0.47 | >99 | 0.5 |
| 6 | 0 | 12 | 25.69 | 0.16 | 0 | 12.5 | 25.51 | 0.11 | 97 | 0.27 |
| 7 | 1 | 8 | 24.98 | 0.02 | 1 | 8.6 | 24.77 | 0.02 | 98 | 0.44 |
| 8 | 1 | 3 | 25.49 | 0.64 | 1 | 4 | 25.59 | 0.59 | >99 | 0.5 |
| 9 | 1 | 2 | 23.92 | 0.97 | 0 | 3 | 23.76 | 0.38 | >99 | 0.5 |
| 10 | 0 | 4 | 23.81 | 0.18 | 0 | 4 | 24.00 | 0.49 | 95 | 0.38 |
| 11 | 0 | 5 | 27.32 | 0.29 | 0 | 6 | 26.26 | 0.43 | >99 | 0.55 |
| 12 | 1 | 7 | 28.58 | 0.2 | 0 | 8 | 28.06 | 0.64 | >99 | 0.5 |
| 13 | 0 | 6 | 26.37 | 0.42 | 0 | 6.3 | 26.15 | 0.44 | >99 | 0.55 |
| 14 | 0 | 4 | 26.32 | 0.66 | 0 | 3 | 26.03 | 0.47 | >99 | 0.55 |
|  |  |  |  | **Mean±SEM = +0.37±0.08** |  |  |  | **Mean±SEM = +0.39±0.05** |  | **Mean±SEM =0.47±0.03** |

**Supplementary 6**

**
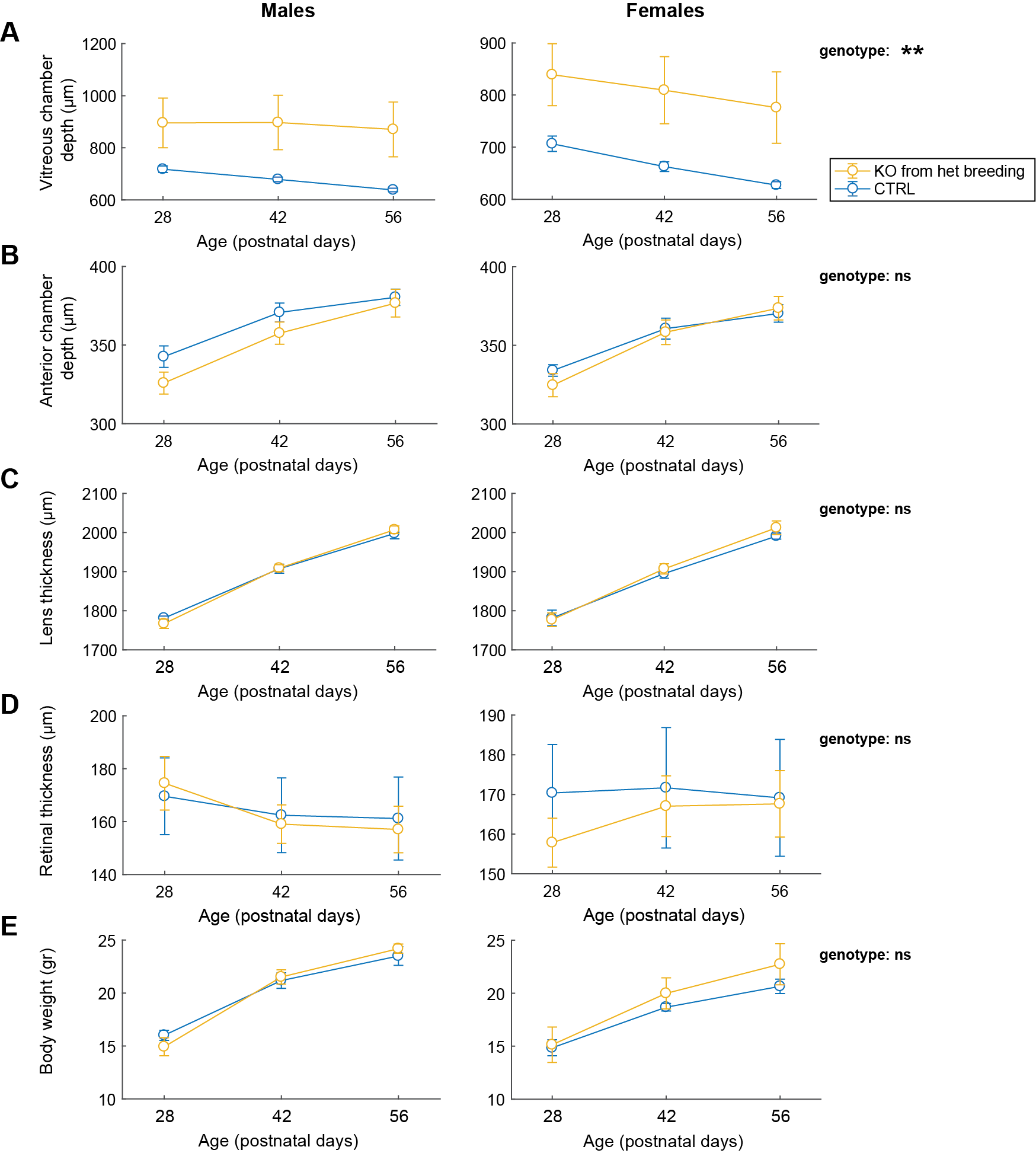
**

***Suppl. 6: Comparison of ocular biometry and body weight between*** ***Foxg1^cre/cre^Lrp2^lox/lox^ (KO) and control (CTRL) littermates.*** *(****A****). KO mice showed statistically significant greater vitreous chamber depth compared to CTRL mice. (****B****) Anterior chamber depth, (****C****) lens thickness, (****D****) retinal thickness, and (****E****) body weight did not show statistically significant differences between KO and CTRL mice. Group sizes: male, KO n=7, CTRL n=9; female KO n=6, CTRL n=8 eyes; ∗∗ p < 0.001–0.01. Linear mixed effects model.*

**Supplementary 7**

**
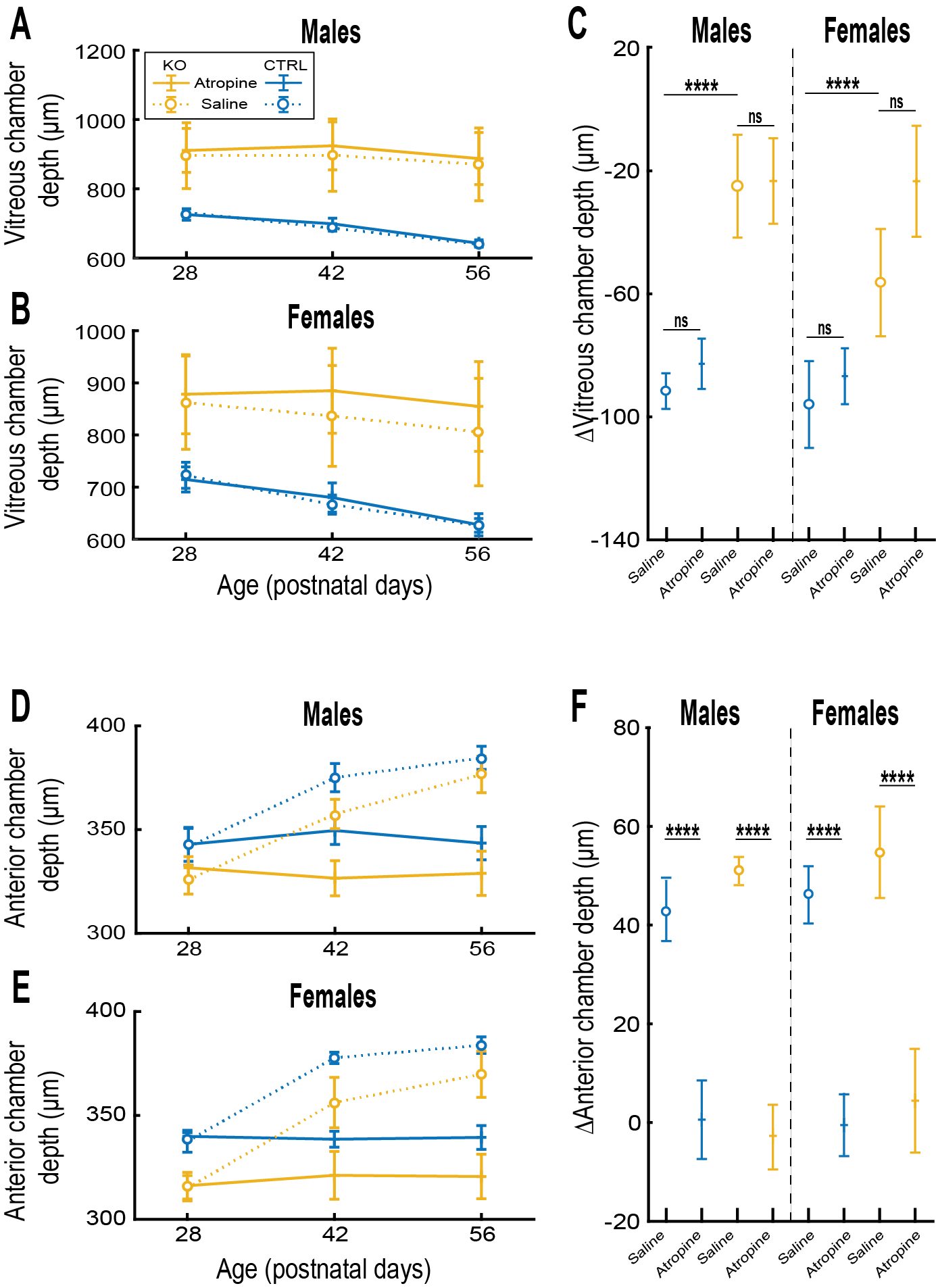
**

***Suppl. 7: Effect of atropine on vitreous chamber depth and anterior chamber depth in Foxg1^cre/cre^Lrp2^lox/lox^ (KO) and control (CTRL) littermates.*** *(****A****). Atropine did not reduce vitreous chamber depth in Lrp2 KO and CTRL male and (****B****) female mice. (****C****). Vitreous chamber depth was generally reducing with age. This reduction was smaller in KO compared to CTRLs. A three-way ANOVA revealed that there was not a statistically significant interaction between the effects of treatment and genotype (See ANOVA tables below). (****D****). Atropine reduced anterior chamber depth in both Lrp2 KO and CTRL male and (****E****) female mice. (****F****). Anterior chamber depth was increased in saline-treated eyes during P28-P56. This increase was nullified when treated with atropine. KO and CTRL mice did not have significant different anterior chamber depths. A three-way ANOVA revealed that there was not a statistically significant interaction between the effects of treatment and genotype (See ANOVA tables below). Group sizes: male, KO n=18, CTRL n=14; female KO n=14, CTRL n=8 mice. ∗ p < 0.01–0.05; ∗∗ p < 0.001–0.01; ∗∗∗ p < 0.0001–0.001; and ∗∗∗∗ p < 0.00001; three-way ANOVA.*

**ANOVA tables of effect of atropine, genotype and gender on ∆vitreous chamber depth and ∆anterior chamber depth during P28-P56.**

∆Vitreous chamber depth:

| **Source** | **Sum Sq.** | **d.f.** | **Mean Sq.** | **F** | **Prob>F** |
| --- | --- | --- | --- | --- | --- |
| **Treatment** | 3372.9 | 1 | 3372.9 | 3.59 | 0.0661 |
| **Genotype** | 24487.4 | 1 | 24487.4 | 26.05 | 0.000010243 |
| **Gender** | 247.2 | 1 | 247.2 | 0.26 | 0.6112 |
| **Treatment x genotype** | 1993.5 | 1 | 1993.5 | 2.12 | 0.1538 |
| **Treatment x gender** | 1.1 | 1 | 1.1 | 0 | 0.9729 |
| **Genotype x gender** | 173 | 1 | 173 | 0.18 | 0.6704 |
| **Error** | 34787.2 | 37 | 940.2 |  |  |
| **Total** | 71337.1 | 43 |  |  |  |

∆Anterior chamber depth:

| **Source** | **Sum Sq.** | **d.f.** | **Mean Sq.** | **F** | **Prob>F** |
| --- | --- | --- | --- | --- | --- |
| **Treatment** | 2585.2 | 1 | 2585.2 | 8.72 | 0.0054 |
| **Genotype** | 2977.9 | 1 | 2977.9 | 10.04 | 0.0031 |
| **Gender** | 12952.2 | 1 | 12952.2 | 43.68 | 0.00000007462 |
| **Treatment x genotype** | 2230.4 | 1 | 2230.4 | 7.52 | 0.0093 |
| **Treatment x gender** | 1530.4 | 1 | 1530.4 | 5.16 | 0.029 |
| **Genotype x gender** | 271.3 | 1 | 271.3 | 0.91 | 0.345 |
| **Error** | 10971.4 | 37 | 296.5 |  |  |
| **Total** | 36206 | 43 |  |  |  |

**Supplementary 8**


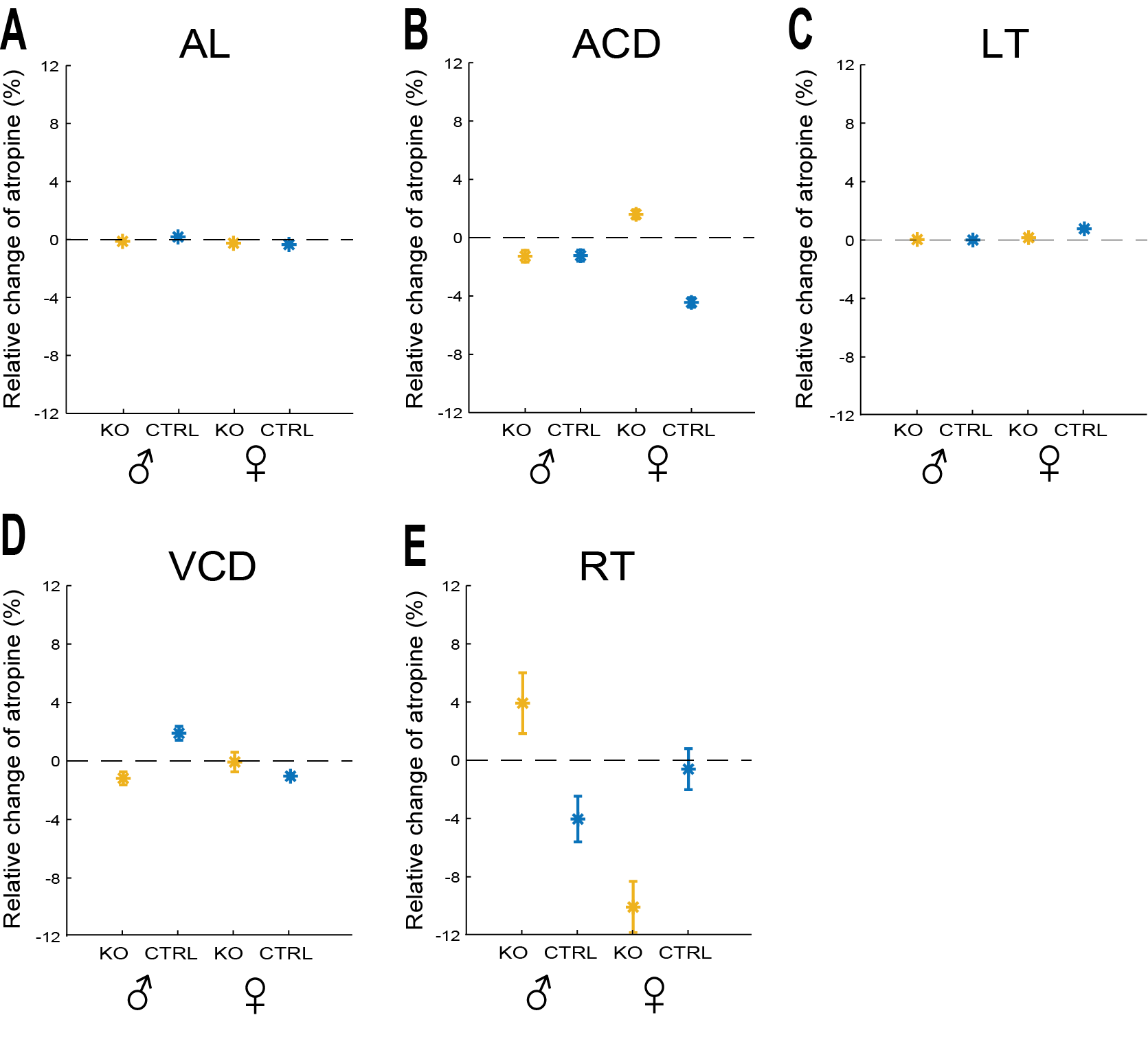


***Suppl. 8: Immediate effect of atropine on ocular biometry, relative to saline, in Foxg1^cre/cre^Lrp2^lox/lox^ (KO) and control (CTRL) male and female mice. Ocular biometry was measured prior and ±4 hours after the first atropine application. The relative change (ocular biometry after – prior/prior * 100%) in atropine-treated eyes minus relative change in saline-treated eyes was calculated.*** *(****A****). Axial length (AL), (****B****) anterior chamber depth (ACD), (****C****) lens thickness (LT), (****D****) vitreous chamber depth (VCD) and (****E****) retinal thickness (RT) were not significantly changed 4 hours after atropine application. Male, KO n=18, CTRL n=14; female, KO n=14, CTRL n=8; two-sample t-test.*

**Supplementary 9**


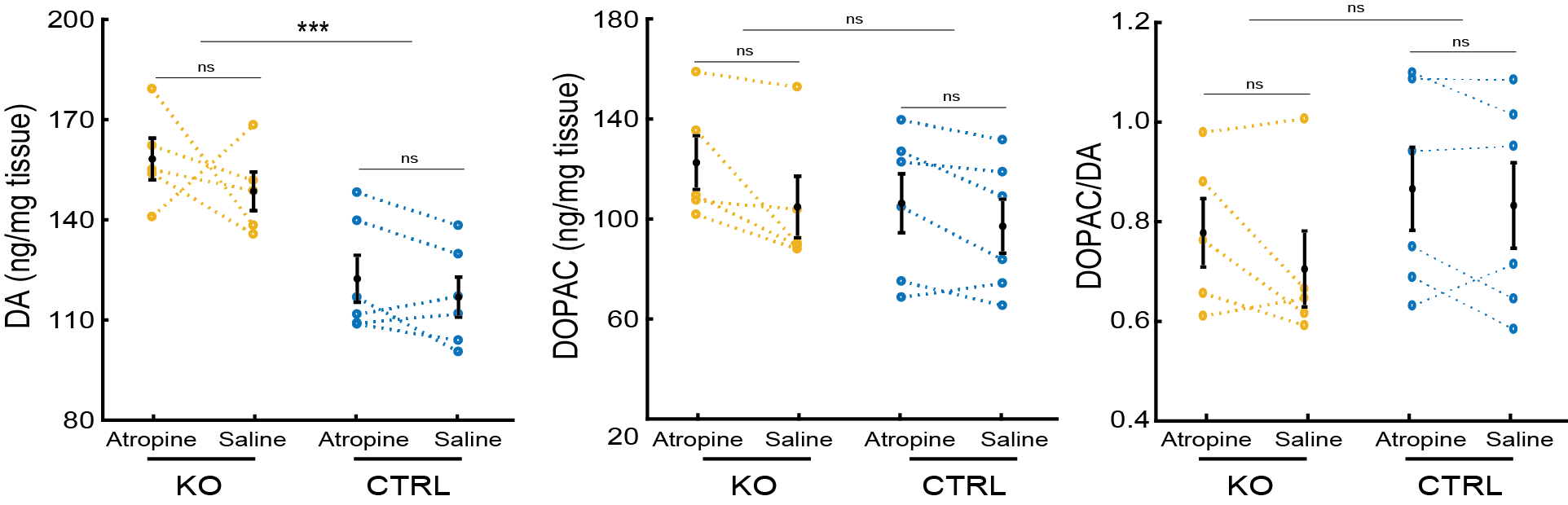
***Suppl. 9: Dopamine (DA) and 3,4-dihydroxyphenylacetic acid (DOPAC) retinal levels and the ratio between DA/DOPAC of Foxg1^cre/cre^Lrp2^lox/lox^ (KO) and control (CTRL) mice, two hours after a single application of atropine, and compared with saline.*** *DA and DOPAC were slightly, albeit nonsignificantly, increased in atropine-treated eyes. DOPAC/DA ratio was not significantly changed by atropine treatment, genotype or gender. Dotted lines between atropine- and saline-treated eyes connect both eyes of one mouse. Group sizes: KO n=5, CTRL n=6 mice; ∗∗∗ p = 0.0001–0.001; three-way ANOVA.*

**ANOVA tables of DA, DOPAC levels and DOPAC/DA ratio two hours after a single application of atropine and saline**

DA level:

| **Source** | **Sum Sq.** | **d.f.** | **Mean Sq.** | **F** | **Prob>F** |
| --- | --- | --- | --- | --- | --- |
| **Treatment** | 295.8 | 1 | 295.78 | 1.19 | 0.2921 |
| **Genotype** | 5781.5 | 1 | 5781.5 | 23.31 | 0.0002 |
| **Gender** | 7.5 | 1 | 7.5 | 0.03 | 0.8643 |
| **Treatment x genotype** | 19.9 | 1 | 19.85 | 0.08 | 0.7811 |
| **Treatment x gender** | 16.8 | 1 | 16.81 | 0.07 | 0.7982 |
| **Genotype x gender** | 284.9 | 1 | 284.92 | 1.15 | 0.3008 |
| **Error** | 3721.1 | 15 | 248.07 |  |  |
| **Total** | 10569.6 | 21 |  |  |  |

DOPAC level:

| **Source** | **Sum Sq.** | **d.f.** | **Mean Sq.** | **F** | **Prob>F** |
| --- | --- | --- | --- | --- | --- |
| **Treatment** | 1024.4 | 1 | 1024.37 | 1.31 | 0.2704 |
| **Genotype** | 571.9 | 1 | 571.85 | 0.73 | 0.406 |
| **Gender** | 6.1 | 1 | 6.13 | 0.01 | 0.9306 |
| **Treatment x genotype** | 114.6 | 1 | 114.62 | 0.15 | 0.7072 |
| **Treatment x gender** | 57.2 | 1 | 57.17 | 0.07 | 0.7906 |
| **Genotype x gender** | 1259.7 | 1 | 1259.75 | 1.61 | 0.2238 |
| **Error** | 11734 | 15 | 782.27 |  |  |
| **Total** | 14876.3 | 21 |  |  |  |

DOPAC/DA ratio:

| **Source** | **Sum Sq.** | **d.f.** | **Mean Sq.** | **F** | **Prob>F** |
| --- | --- | --- | --- | --- | --- |
| **Treatment** | 0.01754 | 1 | 0.01754 | 0.43 | 0.5211 |
| **Genotype** | 0.07061 | 1 | 0.07061 | 1.74 | 0.2071 |
| **Gender** | 0.00015 | 1 | 0.00015 | 0 | 0.9516 |
| **Treatment x genotype** | 0.00296 | 1 | 0.00296 | 0.07 | 0.791 |
| **Treatment x gender** | 0.00833 | 1 | 0.00833 | 0.21 | 0.6571 |
| **Genotype x gender** | 0.02365 | 1 | 0.02365 | 0.58 | 0.4572 |
| **Error** | 0.60928 | 15 | 0.04062 |  |  |
| **Total** | 0.72129 | 21 |  |  |  |

**Supplementary 10**


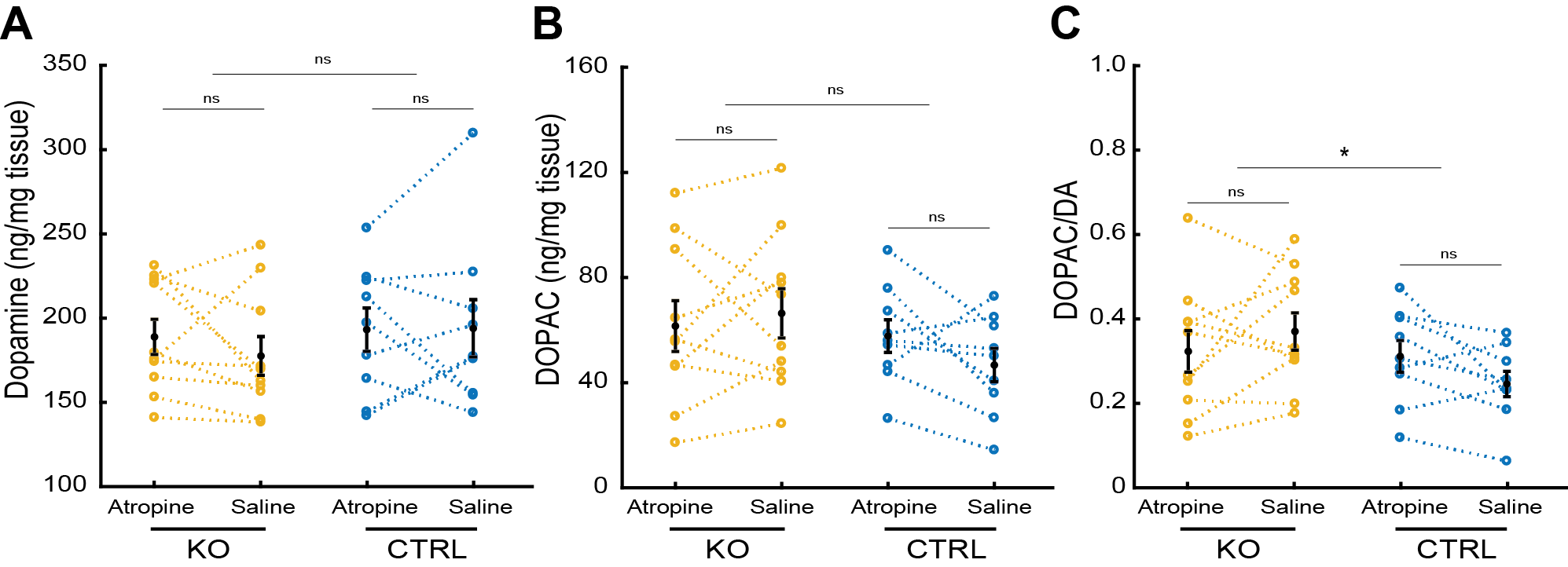


***Suppl. 10: Dopamine (DA) and 3,4-dihydroxyphenylacetic acid (DOPAC) retinal levels of Foxg1^cre/cre^Lrp2^lox/lox^ (KO) and control (CTRL) mice, 24 hours after final atropine application of 4 weeks daily and compared with saline.*** *(****A****). Effect of atropine treatment, KO of Lrp2 gene and gender was not statistically significant changing levels of DA and (****B****). DOPAC in mice at P56. (****C****). Ratio DOPAC/DA was significantly increased in KO mice compared to littermates controls. Main effects of treatment or gender were not statistically significant. Dotted lines between atropine- and saline-treated eyes connect both eyes of one mouse. Group sizes: male, KO n=6, CTRL n=6; female, KO n=4, CTRL n=3 mice; ∗ p < 0.01–0.05; three-way ANOVA.*

**ANOVA tables of DA, DOPAC levels and DOPAC/DA ratio 24 hours after final atropine application of 4 weeks daily**

DA level:

| **Source** | **Sum Sq.** | **d.f.** | **Mean Sq.** | **F** | **Prob>F** |
| --- | --- | --- | --- | --- | --- |
| **Treatment** | 193.5 | 1 | 193.52 | 0.12 | 0.7345 |
| **Genotype** | 2043.3 | 1 | 2043.26 | 1.24 | 0.2747 |
| **Gender** | 15.9 | 1 | 15.89 | 0.01 | 0.9225 |
| **Treatment x genotype** | 360.1 | 1 | 360.06 | 0.22 | 0.6439 |
| **Treatment x gender** | 41.9 | 1 | 41.89 | 0.03 | 0.8745 |
| **Genotype x gender** | 3082.6 | 1 | 3082.62 | 1.87 | 0.1818 |
| **Error** | 51221.5 | 31 | 1652.31 |  |  |
| **Total** | 56090.6 | 37 |  |  |  |

DOPAC level:

| **Source** | **Sum Sq.** | **d.f.** | **Mean Sq.** | **F** | **Prob>F** |
| --- | --- | --- | --- | --- | --- |
| **Treatment** | 62.7 | 1 | 62.67 | 0.1 | 0.7591 |
| **Genotype** | 1743.3 | 1 | 1743.31 | 2.66 | 0.1128 |
| **Gender** | 565.3 | 1 | 565.31 | 0.86 | 0.3599 |
| **Treatment x genotype** | 578.8 | 1 | 578.81 | 0.88 | 0.3543 |
| **Treatment x gender** | 23 | 1 | 22.97 | 0.04 | 0.8526 |
| **Genotype x gender** | 1047.2 | 1 | 1047.25 | 1.6 | 0.2154 |
| **Error** | 20294.1 | 31 | 654.65 |  |  |
| **Total** | 24057.9 | 37 |  |  |  |

DOPAC/DA ratio:

| **Source** | **Sum Sq.** | **d.f.** | **Mean Sq.** | **F** | **Prob>F** |
| --- | --- | --- | --- | --- | --- |
| **Treatment** | 0.00053 | 1 | 0.00053 | 0.04 | 0.8502 |
| **Genotype** | 0.06955 | 1 | 0.06955 | 4.75 | 0.037 |
| **Gender** | 0.02345 | 1 | 0.02345 | 1.6 | 0.2151 |
| **Treatment x genotype** | 0.02948 | 1 | 0.02948 | 2.01 | 0.1658 |
| **Treatment x gender** | 0.00021 | 1 | 0.00021 | 0.01 | 0.9047 |
| **Genotype x gender** | 0.07322 | 1 | 0.07322 | 5 | 0.0326 |
| **Error** | 0.45376 | 31 | 0.01464 |  |  |
| **Total** |  | 37 |  |  |  |
